# Therapeutic Potential of [(AP) _2_M]: A Rationally Designed Polyherbal Formulation for Antidiabetic and Antihyperlipidemic Effects in Alloxan‐Induced Diabetic Mice

**DOI:** 10.1002/fsn3.72520

**Published:** 2026-10-26

**Authors:** Md. Shakil Ahmed, Md. Taohid Hasan, Md. Bismillah, M. Matiar Rahman, Mohammad Ismail Hossain, Masudul Hasan Khan, Mohammad Amirul Islam

**Affiliations:** ^1^ Department of Biochemistry and Molecular Biology, Faculty of Science University of Rajshahi Rajshahi Bangladesh

**Keywords:** acute toxicity, alloxan, antidiabetic, antihyperlipidemic, polyherbal formulation

## Abstract

Medicinal plants are widely used in diabetes management due to their multi‐targeted bioactive constituents. *Phyllanthus emblica* , *Piper nigrum* , *Aegle marmelos* , *Moringa oleifera* , and *Azadirachta indica* possess well‐documented antidiabetic properties, providing a scientific basis for developing a rationally designed polyherbal formulation. This study evaluated the basic phytochemical characterization, antioxidant, antidiabetic, antihyperlipidemic, and preliminary toxicity profiles of a polyherbal formulation (designated as [(AP)_2_M]) in an alloxan‐induced type 1 diabetic mouse model. Polyphenolic (phenolic and flavonoid) contents, antioxidant activity (DPPH & metal‐chelating assays), and α‐amylase and α‐glucosidase inhibitory activities were determined. Acute oral toxicity was assessed according to OECD Guideline 423, with LD_50_ estimation to establish the preliminary toxicity profile and effective dosing. Diabetic mice received the formulation [(AP)_2_M] (30–120 mg/kg) orally for 3 weeks, after which blood glucose, the lipid profile, SGPT, SGOT, and histopathological changes in the liver, kidneys, and pancreas were evaluated. The formulation exhibited substantial phenolic (111.52 ± 0.444 mg GAE/g) and flavonoid (211.30 ± 5.129 mg CAE/g) contents together with strong antioxidant and antidiabetic enzyme inhibitory activity. Acute oral toxicity assessment estimated an LD_50_ of 900 mg/kg (GHS Category 4). At 120 mg/kg, it reduced blood glucose to a level comparable with glibenclamide and significantly improved lipid profile, SGPT, and SGOT levels (*p* < 0.001). Histopathological examination demonstrated improvement in pancreatic, hepatic, and renal tissues. Since the formulation demonstrated promising antioxidant, antihyperglycemic, and antihyperlipidemic activities in a diabetic mouse model, it could be a potential alternative for further investigation as a complementary therapeutic approach for diabetes management.

## Graphical abstract

Development and evaluation of a rationally designed polyherbal formulation, highlighting its phytochemical properties, antioxidant activities, enzyme‐inhibitory effects, and antidiabetic and antihyperlipidemic potential in alloxan‐induced diabetic mice.

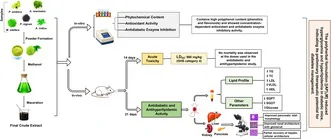

**Abbreviations**
AA: ascorbic acid
ANOVA: analysis of variance
CAE: catechin equivalent
DMSO: dimethyl sulfoxide
DNSA: 3,5‐dinitrosalicylic acid
DPPH: 2,2‐diphenyl‐1‐picrylhydrazyl
DRSA: DPPH radical scavenging activity
EDTA: ethylenediaminetetraacetic acid
FCR: Folin–Ciocalteu reagent
FeCl_2_
: ferrous chloride
Ferrozine: 3‐(2‐pyridyl)‐5,6‐diphenyl‐1,2,4‐triazine‐4′,4″‐disulfonic acid sodium salt
GA: gallic acid
GAE: gallic acid equivalent
GHS: Globally Harmonized System
HDL: high‐density lipoprotein
H&E: hematoxylin and eosin
IC_50_
: half‐maximal inhibitory concentration
LD_50_
: median lethal dose
LDL: low‐density lipoprotein
MCA: metal chelating activity
OECD: Organization for Economic Co‐Operation and Development
pNPG: p‐nitrophenol α‐D‐glucopyranoside
SD: standard deviation
SGOT: serum glutamic oxaloacetic transaminase
SGPT: serum glutamate pyruvate transaminase
TC: total cholesterol
TFC: total flavonoid content
TG: triglycerides
TPC: total phenolic content
VLDL: very low‐density lipoprotein

## 1 Introduction

Diabetes mellitus is a chronic physiological condition defined by persistent hyperglycemia. It is associated with deficiencies in insulin production, insulin action, or both (American Diabetes Association Professional Practice Committee 2021). Globally, it remains one of the fastest‐growing non‐communicable diseases, imposing a substantial clinical, social, and economic burden. According to recent global estimates, approximately 537 million individuals were living with diabetes in 2021, and this number is expected to increase markedly over the coming decades (Ogurtsova et al. 2022).

The pathophysiology of diabetes is multifaceted and involves oxidative stress, impairment of pancreatic β‐cells, and chronic inflammation (DiMeglio et al. 2018). These contribute to microvascular and macrovascular complications affecting the kidneys, liver, cardiovascular system, retina, and peripheral nerves (Paneni et al. 2013). Consequently, effective diabetes management requires not only glycemic control but also interventions capable of mitigating the multiple pathological processes that drive disease progression and associated complications. Although several pharmacological agents—including insulin and oral hypoglycemic drugs—have substantially improved diabetes management, their prolonged use frequently leads to adverse effects, incomplete protection against diabetes‐associated complications, and limited accessibility in many resource‐constrained settings (Ansari et al. 2024; Ahmed and Manirujjaman 2026). Moreover, most conventional therapies primarily target glucose regulation and may not adequately address oxidative stress and other pathological processes involved in the progression of diabetic complications (Ahmed and Manirujjaman 2026). These limitations have stimulated increasing interest in complementary therapeutic strategies capable of simultaneously targeting multiple pathogenic mechanisms while maintaining favorable safety profiles.

Traditional medicine, especially the use of plant‐based remedies, offers a promising avenue for the management of diabetes and other metabolic disorders (Madić et al. 2018). A growing body of evidence indicates that plant‐derived phytochemicals—including phenolics, flavonoids, alkaloids, terpenoids, and other secondary metabolites—exert antidiabetic effects via various mechanisms. These include scavenging reactive oxygen species (ROS), inhibiting carbohydrate‐digesting enzymes, enhancing insulin sensitivity, maintaining pancreatic β‐cell function, regulating inflammatory mediators, and ameliorating diabetes‐associated dyslipidemia (Ansari et al. 2024). These multi‐target biological activities make medicinal plants attractive candidates for developing therapeutic approaches capable of addressing the complex pathophysiology of diabetes rather than a single biochemical pathway.

Building on this concept, polyherbal formulations have received increasing scientific attention. This is because they combine medicinal plants with different phytochemical compositions and pharmacological activities into a single therapeutic intervention (Parasuraman et al. 2014). Numerous experimental studies have reported beneficial antioxidant, antihyperglycemic, and antihyperlipidemic effects of polyherbal formulations in diabetic models (Mahajan et al. 2018; Han et al. 2019; Bera et al. 2010), while recent reviews also highlight their considerable therapeutic potential for metabolic disorders (Palla et al. 2021). Nevertheless, currently available formulations differ substantially in their botanical composition, selection criteria, extraction procedures, phytochemical characterization, and biological evaluation, making direct comparisons difficult and limiting the development of evidence‐based formulations (Palla et al. 2021). Furthermore, although complementary pharmacological actions are frequently proposed as a rationale for polyherbal therapy, such interactions are formulation‐specific and require systematic experimental validation rather than being assumed a priori. Therefore, there remains a need for rationally designed polyherbal formulations supported by clear scientific justification for plant selection and comprehensive biological evaluation using standardized experimental approaches (Palla et al. 2021).

In the present study, five medicinal plants, *Phyllanthus emblica* L., *Piper nigrum* L., *Aegle marmelos* (L.) Corrêa, *Moringa oleifera* Lam., and *Azadirachta indica* A. Juss., were selected based on three principal criteria: (i) their well‐documented traditional use in diabetes management, (ii) substantial experimental evidence supporting antidiabetic activity, and (iii) their reported ability to target different pathological processes involved in diabetes (Ahmed, Hasan, Ahmmed, et al. 2025). Specifically, published evidence indicates that these plants modulate distinct yet interconnected biological pathways involved in diabetes pathogenesis. For example, *P. emblica* exhibits potent antioxidant activity, suppresses α‐amylase and α‐glucosidase, enhances insulin signaling, and improves glucose homeostasis (Prananda et al. 2023), while *A. marmelos* suppresses carbohydrate‐digesting enzymes and alleviates oxidative stress, thereby reducing postprandial glucose absorption and diabetes‐associated complications (Venkatesan et al. 2024). In contrast, *A. indica* has been associated with antioxidant activity, improved insulin responsiveness, and protection of pancreatic β‐cells (Yarmohammadi et al. 2021), whereas *P. nigrum* —largely through its bioactive alkaloid piperine—improves insulin sensitivity by attenuating metabolic inflammation and oxidative stress (Wang et al. 2023; Liu et al. 2020). *M. oleifera* has demonstrated antihyperglycemic, hypolipidemic, antioxidant, and insulin‐sensitizing activities while also modulating inflammatory responses associated with metabolic dysfunction (Louisa et al. 2022). Collectively, these reported biological activities provide a pharmacological rationale for investigating a formulation designed to target multiple mechanisms contributing to diabetes progression.

In our previous study, methanolic extracts of the same five medicinal plants were comparatively evaluated under standardized identical experimental conditions for their in vitro phytochemical profile, antioxidant potential, antidiabetic‐related enzyme inhibitory activity, and cytotoxicity (Ahmed, Hasan, Ahmmed, et al. 2025). That investigation revealed considerable variation in their biological activities and provided a comparative evidence base for selecting medicinal plants with promising antidiabetic potential for subsequent formulation development. Because these plants have been reported to target different pathological processes associated with diabetes (Prananda et al. 2023; Venkatesan et al. 2024; Yarmohammadi et al. 2021; Wang et al. 2023; Liu et al. 2020; Louisa et al. 2022), integrating them into a single formulation was considered a rational strategy to investigate whether a multi‐component approach could provide a broader pharmacological profile than individual plant extracts alone. Therefore, the present study extends our previous work by developing a rationally designed polyherbal formulation and evaluating its (i) polyphenolic profiling, antioxidant capacity, and in vitro α‐amylase and α‐glucosidase inhibitory activities; (ii) acute oral toxicity to establish its preliminary toxicity profile; (iii) antihyperglycemic and antihyperlipidemic properties in an alloxan‐induced type 1 diabetic mouse model; and (iv) effects on biochemical markers and histopathological alterations in the pancreas, kidneys, and liver to provide a comprehensive assessment of its biological activity.

Although polyherbal formulations containing one or more of these medicinal plants have previously been reported (Brahma et al. 2024), to the best of our knowledge, this study represents the first comprehensive in vitro and in vivo evaluation of the present rationally designed polyherbal formulation (designated as [(AP)_2_M]) developed from prior comparative screening of its constituent medicinal plants. By integrating comparative in vitro evidence with comprehensive in vivo evaluation, the present study provides experimental evidence supporting the rational development of polyherbal formulations for diabetes management while establishing a foundation for future mechanistic and translational investigations.

## 2 Material and Methods

### 2.1 Chemicals

Acarbose, alloxan monohydrate, catechin, ferrozine (3‐(2‐pyridyl)‐5,6‐diphenyl‐1,2,4‐triazine‐4′,4″‐disulfonic acid sodium salt), gallic acid, and DPPH (2,2‐diphenyl‐1‐picrylhydrazyl) were sourced from Sigma‐Aldrich, USA. Ascorbic acid (AA) was obtained from Scharlau, Spain. FCR (Folin–Ciocalteu's phenol reagent) was purchased from Merck (Darmstadt, Germany). pNPG (p‐nitrophenol α‐D‐glucopyranoside) was obtained from Sigma Chemical Co. (St. Louis, MO, USA). Commercial assay kits for total cholesterol (TC) (Erba Diagnostics, Mannheim, Germany), triglycerides (TG), SGPT (serum glutamate pyruvate transaminase), and SGOT (serum glutamic oxaloacetic transaminase) (Elzzad Biosystem, Singapore), as well as LDL (low‐density lipoprotein) and HDL (high‐density lipoprotein) (Cromatest, Linear Chemicals, Spain), were obtained from the respective manufacturers. Analytical‐grade reagents were employed throughout the experiments, with all solutions prepared using deionized water.

### 2.2 Plants Collection and Extract Preparation

The leaves of *A. indica* , *M. oleifera* , and *A. marmelos* were obtained from the areas surrounding the University of Rajshahi, while the fruits of *P. nigrum* and *P. emblica* were brought from a local market in Rajshahi, Bangladesh between January 22 and February 13, 2024. The plant samples were taxonomically identified and authenticated by Mr. Arshed Alom, Herbarium, Department of Botany, University of Rajshahi, Bangladesh, on February 22, 2024. Voucher specimens were deposited in the Department of Botany Herbarium, University of Rajshahi, under voucher numbers 14 ( *P. emblica* ), 84 ( *P. nigrum* ), 103 ( *A. marmelos* ), 39 ( *M. oleifera* ), and 40 ( *A. indica* ).

Collected plant samples were thoroughly cleaned, dried in the shade, and milled to a fine powder. The polyherbal formulation was prepared using 17.70% *P. emblica* fruit powder, 23.92% *P. nigrum* fruit powder, 20.38% *A. marmelos* leaf powder, 18.90% *M. oleifera* leaf powder, and 19.10% *A. indica* leaf powder. The initial formulation proportions were determined using a normalized inverse fourth‐root weighting scheme, based on the total phenolic content (TPC) of the individual plant materials, following the findings of our preliminary in vitro study (Ahmed, Hasan, Ahmmed, et al. 2025). The reported TPC values were 192.09 ± 2.08, 69.37 ± 2.20, 27.48 ± 1.35, 99.37 ± 0.93, and 83.81 ± 1.67 mg GAE/g for *P. emblica* , *A. marmelos* , *P. nigrum* , *M. oleifera* , and *A. indica* , respectively. The TPC‐derived proportions were subsequently subjected to a slight adjustment based on the comparative toxicity findings reported in the preliminary study (Ahmed, Hasan, Ahmmed, et al. 2025), as detailed in the Appendix S1. Polyphenolic content (TPC) was selected as the primary criterion because it closely reflected the antioxidant capacity and α‐amylase and α‐glucosidase inhibitory properties of the individual plant extracts reported in the preliminary investigation. To achieve a balanced phytochemical composition, plant materials with relatively higher polyphenolic contents were incorporated at lower proportions, thereby preventing any single phytochemically rich component from dominating the formulation while maintaining the contribution of bioactive constituents from all five medicinal plants.

The powdered mixture was extracted with methanol at a 1:5 (w/v) ratio and kept for maceration at ambient temperature for 2 weeks. After maceration, Whatman filter paper No. 41 was utilized to purify the extracts, and the solvent was removed utilizing a rotary evaporator under vacuum conditions. The resulting formulation extract was preserved at 4°C for subsequent experimental use.

#### 2.2.1 Formulation Designation

Throughout this study, the polyherbal formulation is referred to as the unified designation [(AP)_2_M], where the letters correspond to the first letter of the plant genera: A represents *A. indica* and *A. marmelos* , P represents *P. emblica* and *P. nigrum* , and M represents *M. oleifera* .

### 2.3 Estimation of Total Phenolic Content (TPC)

TPC in the extracts was determined following a modified method of Miliauskas et al. (2004). For the assay, 0.3 mL of extract was combined with 2.25 mL of FCR, which had been diluted 1:10 (v/v) with deionized water, along with 2 mL of a 7.5% (w/v) Na_2_CO_3_ solution. This mixture was kept in the dark at 25°C for 1 h. The absorbance was then recorded at 760 nm, and the TPC value was calculated as gallic acid equivalents (GAE) per gram of dry extract.

### 2.4 Estimation of Total Flavonoid Content (TFC)

TFC was measured by a modified procedure reported by Zhishen et al. (1999). In this protocol, 0.5 mL of the test sample was combined with 0.15 mL of 5% (w/v) sodium nitrite solution and 2.25 mL of deionized water. After allowing the mixture to stand for 5 min, 0.3 mL of 10% (w/v) aluminum chloride solution was added. Following an additional incubation period of 6 min, 1 mL of sodium hydroxide (1 M) was incorporated, and the absorbance was recorded at 510 nm. Catechin was utilized as the calibration standard, with the results reported as catechin equivalents (CAE) per gram of dry weight sample.

### 2.5 DPPH Free Radical Scavenging Activity (DRSA)

The DPPH radical‐scavenging capacity of the sample was determined following the method outlined by Brand‐Williams et al. (1995). In brief, 1 mL of the extract or standard (AA) solutions at varying concentrations was added to 3 mL of 0.1 mM DPPH solution prepared in methanol. The contents were mixed thoroughly and then kept in the dark at 25°C for 30 min. The absorbance was recorded at 517 nm using a UV–visible spectrophotometer. DPPH radical‐scavenging activity (%) was calculated using the following equation:

(1) $$\text{Percent inhibition}\;\left(\%\right)=\frac{A_{s_{0}}-A_{s}\;}{A_{s_{0}}}\times 100$$

Here,

$A_{s}$ = the absorbance of the sample,

$A_{s_{0}}$ = the absorbance of the control.

### 2.6 Metal Chelating Activity (MCA)

This procedure was previously reported by Dinis et al. (1994). A 2 mL aliquot of the sample—prepared at concentrations ranging from 20 to 200 μg/mL—was combined with 0.05 mL of FeCl_2_ solution (2 mM). Subsequently, 0.2 mL of ferrozine solution (5 mM) was added to start the reaction. After incubation at 25°C for 10 min, the absorbance of the mixture was measured at 562 nm. Ethylenediaminetetraacetic acid (EDTA) was used as the standard. The percentage of chelating activity was estimated using the formula provided earlier (Equation 1).

### 2.7 α‐Amylase Inhibitory Assay

This assay was conducted using a modified protocol based on the procedure reported by Kazeem and Ashafa (2015). In this protocol, 0.5 mL of 200 mM phosphate buffer (pH 6.9) supplemented with 6 mM NaCl was mixed with 0.5 mL of the test samples at each concentration—ranging from 25 to 250 μg/mL—in test tubes. An equal volume (0.5 mL) of 2 U/mL α‐amylase enzyme solution was then added. Subsequently, 0.5 mL of 1% starch solution was added to start the reaction. The mixture was allowed to stand at 25°C for 15 min. To terminate the enzymatic process, DNSA reagent (0.5 mL) was added, and the contents were subsequently boiled for 5 min. Upon cooling to room temperature, each mixture was diluted with deionized water (2 mL). Acarbose served as the reference standard, and the final absorbance was read at 540 nm. The % α‐amylase inhibition was determined using the Equation (1) described above.

### 2.8 α‐Glucosidase Inhibitory Assay

This method was carried out following a modified method adapted from Yao et al. (2013). In this assay, 0.5 mL of either the test extract or standard sample was combined with 1 mL of phosphate buffer (50 mM, pH 6.9). Next, 0.05 mL of 2 U/mL α‐glucosidase enzyme solution was combined with the mixture. After incubating the mixture at 37°C for 10 min, 0.05 mL of 70 mM pNPG was introduced as the substrate. The mixture was then incubated for an additional 30 min at the same temperature. To end the reaction, 2 mL of sodium carbonate (0.5 M) was added, and the absorbance was measured at 405 nm. Acarbose was employed as the standard inhibitor. The % α‐glucosidase inhibition was determined using the Equation (1).

### 2.9 Oral Acute Toxicity Assessment

The acute oral toxicity assessment was performed, following the recommendations of the guideline from the Organization for Economic Co‐Operation and Development (OECD 2002) 423, with slight changes to permit estimation of the median lethal dose (LD_50_) (Chinedu et al. 2013). Healthy adult female mice (Swiss albino) were allocated randomly into five groups (5 mice per group) and were treated with the test substance via oral gavage at doses from 100 to 1600 mg/kg body weight. The required formulation doses were dissolved in DMSO and diluted with distilled water to a final volume of 0.2 mL, with the DMSO concentration varying from 0.125% to 0.5% depending on the required dose. The mice were observed for 14 days to monitor mortality and visible behavioral abnormalities—such as traction, salivation, hair erection, tremors, lacrimation, loss of appetite, and diarrhea—along with other potential toxicity signs. The LD_50_ was estimated from the mortality data using the arithmetic method of Karber (Chinedu et al. 2013), according to the following equation:

(2) $${\mathrm{LD}}_{50}={\mathrm{LD}}_{100}-\frac{\sum \left(a\times b\right)}{n}$$

Here,

LD_100_ = lowest dose producing 100% mortality,

*a* = dose interval,

*b* = average mortality between adjacent doses,

*n* = number of animals in each group.

### 2.10 In Vivo Antidiabetic Experiment

#### 2.10.1 Animals Used in the Study

Adult female mice (Swiss albino), weighing 25–30 g, were selected for the study. The mice were bred and maintained in the Department of Biochemistry and Molecular Biology's Animal House at the University of Rajshahi, Bangladesh. Before the investigation, the mice were acclimatized for 7 days under standard laboratory conditions in a properly ventilated condition at 23°C–25°C, with unrestricted access to standard pellet feed and water ad libitum.

#### 2.10.2 Induction of Experimental Diabetes and Study Design

Following a 12‐h fasting period, experimental diabetes was induced through a single intraperitoneal administration of alloxan monohydrate (135 mg/kg body weight), thereby establishing an alloxan‐induced type 1 diabetic mouse model. The animals were subsequently maintained on their regular laboratory diet with water provided ad libitum. Blood glucose levels were initially assessed 48 h after alloxan administration; however, as several animals exhibited fasting blood glucose levels < 200 mg/dL, the mice were monitored for an additional 24 h. Diabetes was confirmed 72 h after alloxan administration, and mice exhibiting fasting blood glucose levels ≥ 200 mg/dL were designated as diabetic and subsequently enrolled in the study. Treatment with the polyherbal formulation [(AP)_2_M] or glibenclamide was initiated on the day following confirmation of diabetes.

A total of six groups were established, each consisting of six mice. Group I was used as the normal control, while Group II was designated as the diabetic control, with both receiving only saline. Group III was given glibenclamide (5 mg/kg body weight), and Groups IV, V, and VI were treated with the polyherbal formulation at doses of 30, 60, and 120 mg/kg body weight, respectively. The treatment doses were selected based on the results of the acute oral toxicity study. The required doses of the formulation were dissolved in DMSO and diluted with distilled water to a final volume of 0.2 mL containing 0.125% DMSO before administration. All treatments were administered orally by gavage once daily for 3 weeks (Ahmed, Hasan, Alam, et al. 2025).

#### 2.10.3 Blood Glucose Level Assessment

Blood glucose concentrations were measured using the partial tail amputation technique by obtaining a blood sample from the tail vein. The collected blood was analyzed using glucose test strips with a OneTouch electronic glucometer. Following blood collection, the tail was disinfected with an ethanol pad to minimize the risk of infection. Glucose measurements were performed on Days 1, 7, 14, and 21 throughout the 21‐day experimental period. Before each measurement, all experimental mice were fasted for at least 12 h.

#### 2.10.4 Body Weight Measurement

Body weights of the experimental mice were measured before the initiation of the treatment and subsequently on Days 1, 7, 14, and 20 to monitor weight fluctuations throughout the study period.

#### 2.10.5 Collecting and Preparing the Organs and Blood Samples

After the 21‐day experimental period, the mice were subjected to an overnight fast (8–10 h). The mice were anesthetized using 4% chloroform and sacrificed, and blood was drawn via the inferior vena cava and held in sterile tubes for 30 min to allow coagulation. The blood samples underwent centrifugation at 3000 rpm for 10 min to collect serum from the clotted blood. The mice's organs were carefully removed and washed with phosphate‐buffered saline. They were then placed in a 10% formalin solution in a sterile organ collection container and stored for subsequent use.

#### 2.10.6 Biochemical Examination

The serum samples were analyzed for various biochemical parameters, including lipid biomarkers such as TC, TG, LDL, and HDL, as well as hepatic enzymes, specifically SGPT and SGOT. Analyses were conducted utilizing standard diagnostic kits obtained from commercial sources, in accordance with the manufacturer's protocols. The VLDL values were calculated from the experimental TG values using the formula: $\text{VLDL}=\frac{\mathrm{TG}}{5}$.

#### 2.10.7 Histopathologic Analysis

After the experiment, the mice's pancreas, kidney, and liver were excised and submerged in a 10% buffered saline solution for histological assessment. Tissue samples were processed and subjected to hematoxylin and eosin (H&E) staining according to established protocols. The morphological alterations in pancreatic, renal, and hepatic tissues were visualized under a light microscope on albumenized glass slides at 40× magnification (Fischer et al. 2008).

### 2.11 Statistical Analysis

The results are expressed as mean ± standard deviation (SD). Graphical representations and statistical analyses were performed using GraphPad Prism Software (version 9.5) and R software (version 2024), respectively. To evaluate statistical significance, one‐way analysis of variance (ANOVA) followed by Dunnett's post hoc test was performed, with *p* < 0.05 indicating statistical significance.

## 3 Results

### 3.1 Polyphenolic Contents

Table 1 shows the polyphenolic (phenolic and flavonoid) contents of the polyherbal formulation. The formulation contained 111.52 ± 0.444 mg GA/g of phenolic compounds and 211.30 ± 5.129 mg CA/g of flavonoid compounds.

**TABLE 1:** Polyphenolic contents of the polyherbal formulation.

Sample	Phenolics^a^	Flavonoids^b^
[(AP)_2_M]	111.52 ± 0.444	211.30 ± 5.129

*Note:* Data are presented as the mean ± SD of three technical replicates. The superscript letters a and b represent gallic acid equivalent (GAE) and catechin equivalent (CAE), respectively, and are reported in mg/g of dry extract.

### 3.2 In Vitro Antioxidant Capacity

Both the DPPH and MCA assays demonstrated a concentration‐dependent radical scavenging effect for the polyherbal formulation and the standards (AA and EDTA). The IC_50_ values were recorded as 26.70 ± 0.933 μg/mL for the formulation and 12.73 ± 0.354 μg/mL for AA in the DPPH assay, while in the metal chelating test, EDTA showed an IC_50_ of 4.58 ± 0.200 μg/mL, and the polyherbal formulation exhibited a value of 29.11 ± 0.509 μg/mL. The formulation extract showed moderate activity compared to the standard AA and EDTA, as illustrated in Figure 1.

**FIGURE 1:**
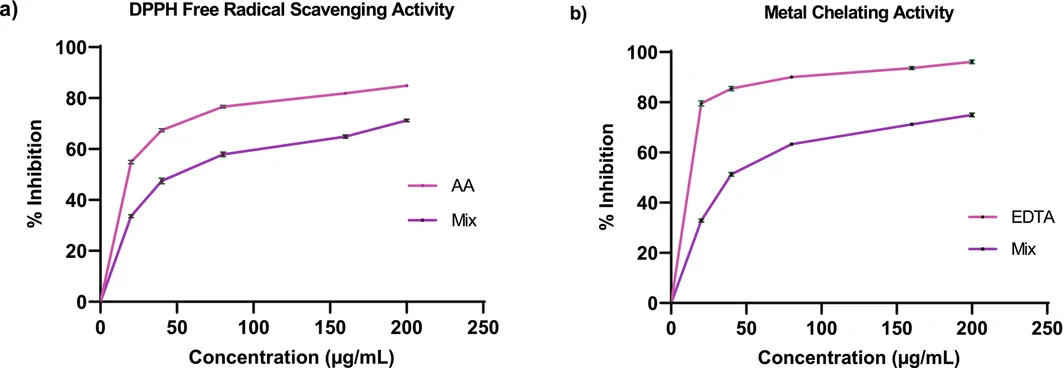
In vitro antioxidant activity of the polyherbal formulation extract. (a) DPPH free radical scavenging activity and (b) Metal chelating activity. Values are expressed as mean ± SD of three technical replicates (*n* = 3). AA, ascorbic acid; EDTA, ethylenediaminetetraacetic acid; Mix, polyherbal formulation.

### 3.3 In Vitro Antidiabetic Activity

Figure 2 illustrates the inhibitory effects of the polyherbal formulation and the standard acarbose on α‐amylase and α‐glucosidase. Both the formulation and acarbose exhibited concentration‐dependent inhibition. At 250 μg/mL, the inhibitory effects were 64.41% ± 0.246% for acarbose and 58.28% ± 0.511% for the polyherbal formulation in the α‐amylase inhibitory assay, and 93.77% ± 0.226% and 82.53% ± 0.470%, respectively, in the α‐glucosidase inhibitory assay.

**FIGURE 2:**
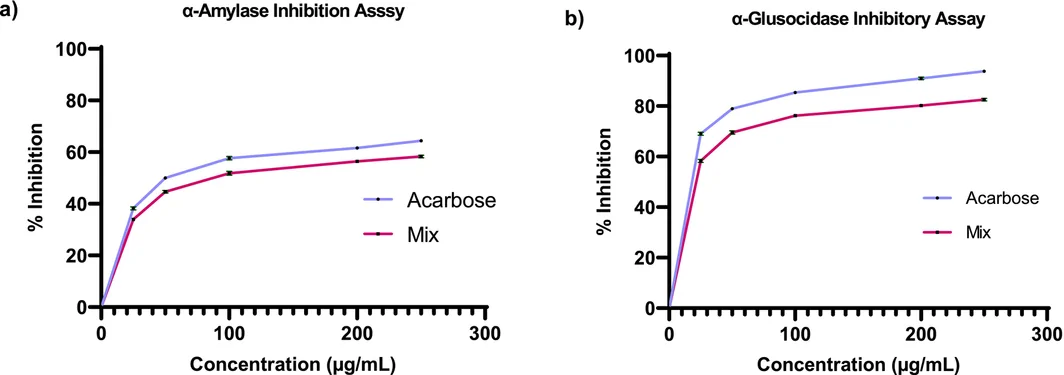
In vitro antidiabetic activity of the polyherbal formulation extract. (a) α‐Amylase inhibitory activity and (b) α‐Glucosidase inhibitory activity. Values are expressed as mean ± SD of three technical replicates (*n* = 3). Mix, polyherbal formulation.

### 3.4 Acute Toxicity Assessment

The LD_50_ of the polyherbal formulation, estimated using the arithmetic method of Karber from the observed mortality data, was approximately 900 mg/kg body weight (see Appendix S1). According to the OECD Guideline 423 acute oral toxicity classification criteria, this value corresponds to Globally Harmonized System (GHS) Category 4 (300–2000 mg/kg), indicating moderate acute oral toxicity (OECD 2002). No mortality or abnormal clinical signs were detected at 100 mg/kg, while animals receiving 200 mg/kg showed only a slight reduction in food intake. At higher doses (400–1600 mg/kg), dose‐dependent toxic manifestations—including reduced food intake, diarrhea, and lethargy—were observed, accompanied by increasing mortality (see Table 2).

**TABLE 2:** Mortality and clinical observations of mice following acute oral administration of the polyherbal formulation.

Dose (mg/kg)	Mortality (dead/total)	Clinical observations
100	0/5	No abnormal clinical signs.
200	0/5	Slight reduction in food intake was observed in some animals during the observation period; no other abnormal signs were noted.
400	1/5	Mild reduction in food intake, and diarrhea were observed in some animals prior to mortality.
800	2/5	Reduced activity (lethargy), decreased food intake, and diarrhea were observed in some animals prior to mortality.
1600	5/5	Marked lethargy, reduced food intake, diarrhea, and subsequent mortality were observed in all animals.

### 3.5 In Vivo Antidiabetic Experiment

#### 3.5.1 Effect on Blood Glucose

The polyherbal formulation [(AP)_2_M], administered at doses of 30, 60, and 120 mg/kg, significantly lowered (*p* < 0.001) blood glucose concentrations in diabetic mice in a dose‐responsive pattern. The raw experimental values are presented in Table S1, while the multiple comparisons—diabetic vs. glibenclamide, low‐, medium‐ and high‐dose groups—are shown in Figure 3. Notably, the glucose‐lowering effect observed in the 120 mg/kg group was comparable to the results seen in the glibenclamide‐treated group, with similar mean values between the two.

**FIGURE 3:**
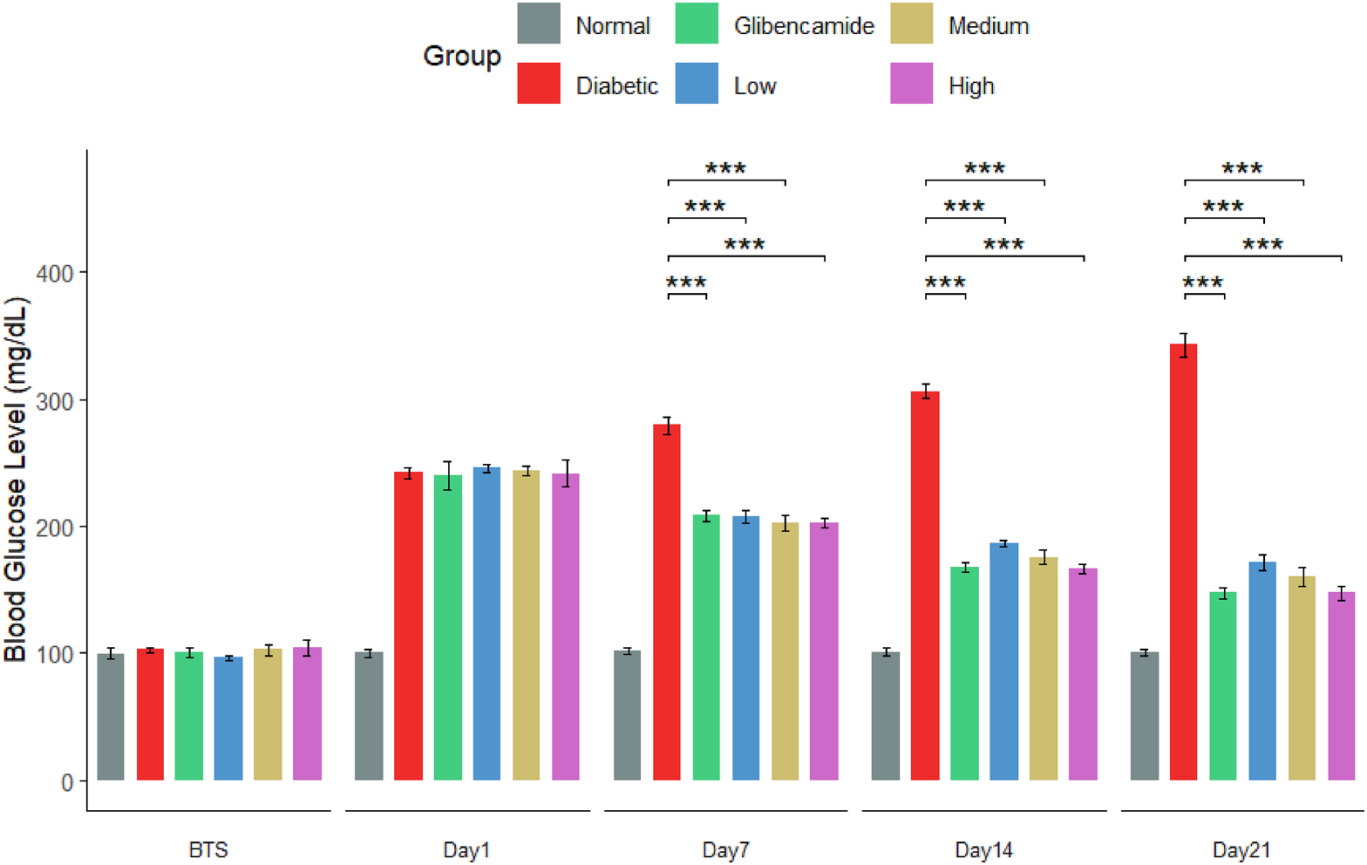
Change in blood glucose concentration during the investigation. Data are expressed as mean ± SD, with *n* = 6 mice per group. Statistical significance was assessed using one‐way ANOVA followed by Dunnett's post hoc test, with *p* < 0.05 indicating statistical significance. BTS, before treatment starts. Here, ***p < 0.001.

#### 3.5.2 Changes in Body Weight

Significant increases in body weight (*p* < 0.05, *p* < 0.01, and *p* < 0.001) were noted in alloxan‐induced diabetic mice following 21 days of treatment with the methanolic extracts of the polyherbal formulation. The experimental data are shown in Table S2, and the comparisons between the Diabetic group and the treatment groups are presented in Figure 4.

**FIGURE 4:**
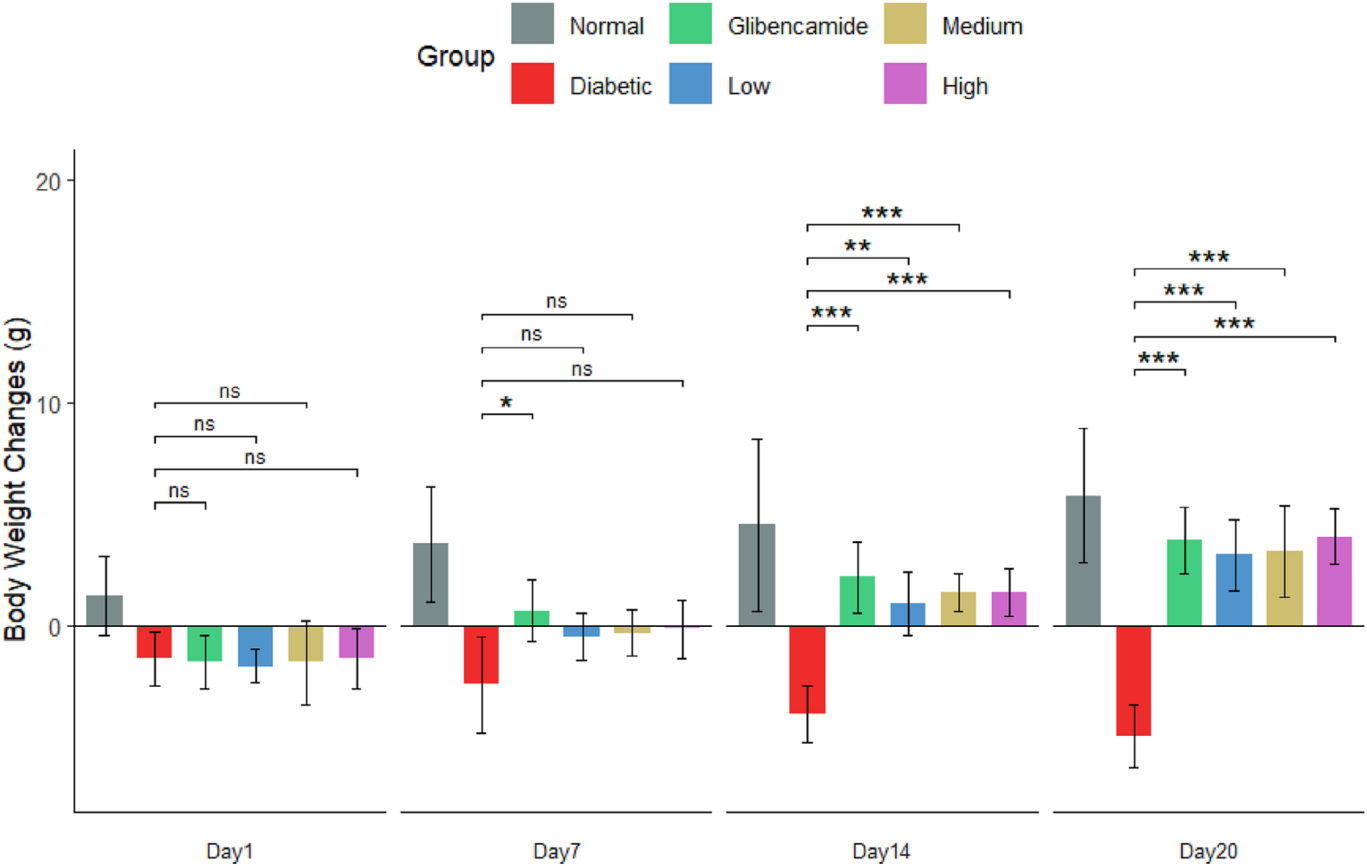
Body weight changes during the investigation. Values are reported as mean ± SD, with *n* = 6 mice per group. Statistical significance was assessed using one‐way ANOVA followed by Dunnett's post hoc test, with *p* < 0.05 indicating statistical significance. Here, *p < 0.05; **p < 0.01; and ***p < 0.001.

#### 3.5.3 Lipid Profile Parameters

Alloxan administration caused significant changes in the lipid parameters among diabetic mice in comparison with the control group. Treatment with the methanolic extract of the polyherbal formulation, however, led to a significant decrease (*p* < 0.001) in lipid levels, restoring them toward normal ranges. Table 3 presents the raw data together with the results of statistical comparisons between the Diabetic group and all other groups.

**TABLE 3:** Effect of the polyherbal formulation on lipid profile and serum biochemical parameters.

Groups	TG (mg/dL)	TC (mg/dL)	LDL (mg/dL)	HDL (mg/dL)	VLDL (mg/dL)	SGPT (U/L)	SGOT (U/L)
Normal	122.53 ± 7.57^a^	147.57 ± 2.59^a^	49.00 ± 2.66^a^	73.71 ± 5.11^a^	24.51 ± 1.51^a^	21.26 ± 0.74^a^	19.38 ± 0.46^a^
Diabetes	217.44 ± 6.32	234.85 ± 6.51	162.68 ± 6.58	28.50 ± 5.34	43.49 ± 1.26	61.88 ± 1.57	56.16 ± 1.89
Glibenclamide	105.74 ± 5.36^a^	144.73 ± 4.74^a^	63.26 ± 4.77^a^	65.72 ± 8.06^a^	21.15 ± 1.07^a^	34.43 ± 0.53^a^	25.06 ± 0.33^a^
Low dose	118.52 ± 6.92^a^	164.47 ± 4.38^a^	88.52 ± 4.40^a^	52.52 ± 3.29^a^	23.70 ± 1.38^a^	38.40 ± 0.42^a^	32.24 ± 0.75^a^
Medium dose	112.98 ± 2.58^a^	157.15 ± 3.15^a^	79.08 ± 5.12^a^	55.19 ± 3.28^a^	22.60 ± 0.52^a^	36.30 ± 1.00^a^	28.72 ± 1.32^a^
High dose	103.67 ± 6.08^a^	145.11 ± 4.18^a^	62.13 ± 4.29^a^	62.01 ± 4.85^a^	20.73 ± 1.22^a^	29.25 ± 1.58^a^	26.28 ± 0.33^a^

*Note:* Values are reported as mean ± SD, with *n* = 6 mice per group. Statistical significance was assessed using one‐way ANOVA followed by Dunnett's post hoc test, with the diabetic control group used as the reference group. *p* < 0.05 was considered statistically significant.

Abbreviations: HDL, high‐density lipoproteins; LDL, low‐density lipoproteins; SGOT, serum glutamic oxaloacetic transaminase; SGPT, serum glutamate pyruvate transaminase; TC, total cholesterol; TG, triglycerides; VLDL, very low‐density lipoproteins.

^a^ *p* < 0.001.

#### 3.5.4 Serum Profile Parameters

SGPT and SGOT levels were significantly increased in alloxan‐induced diabetic mice relative to the control group. However, treatment with the polyherbal extract and glibenclamide resulted in a notable reduction in these enzyme levels, demonstrating improvement. The experimental values are provided in Table 3 with detailed comparisons between the diabetic group and all other groups.

#### 3.5.5 Histopathological Analysis

According to the pancreatic histopathological observation (Figure 5), the diseased mice's pancreatic islets degenerated, showing smaller cell clusters and fewer islets in general. The islets appeared relatively preserved in the normal control group. Treatment with glibenclamide, low, medium, and high doses of the polyherbal formulation groups showed improvements in the pancreatic islet morphology, with varying degrees of recovery from the observed degeneration.

**FIGURE 5:**
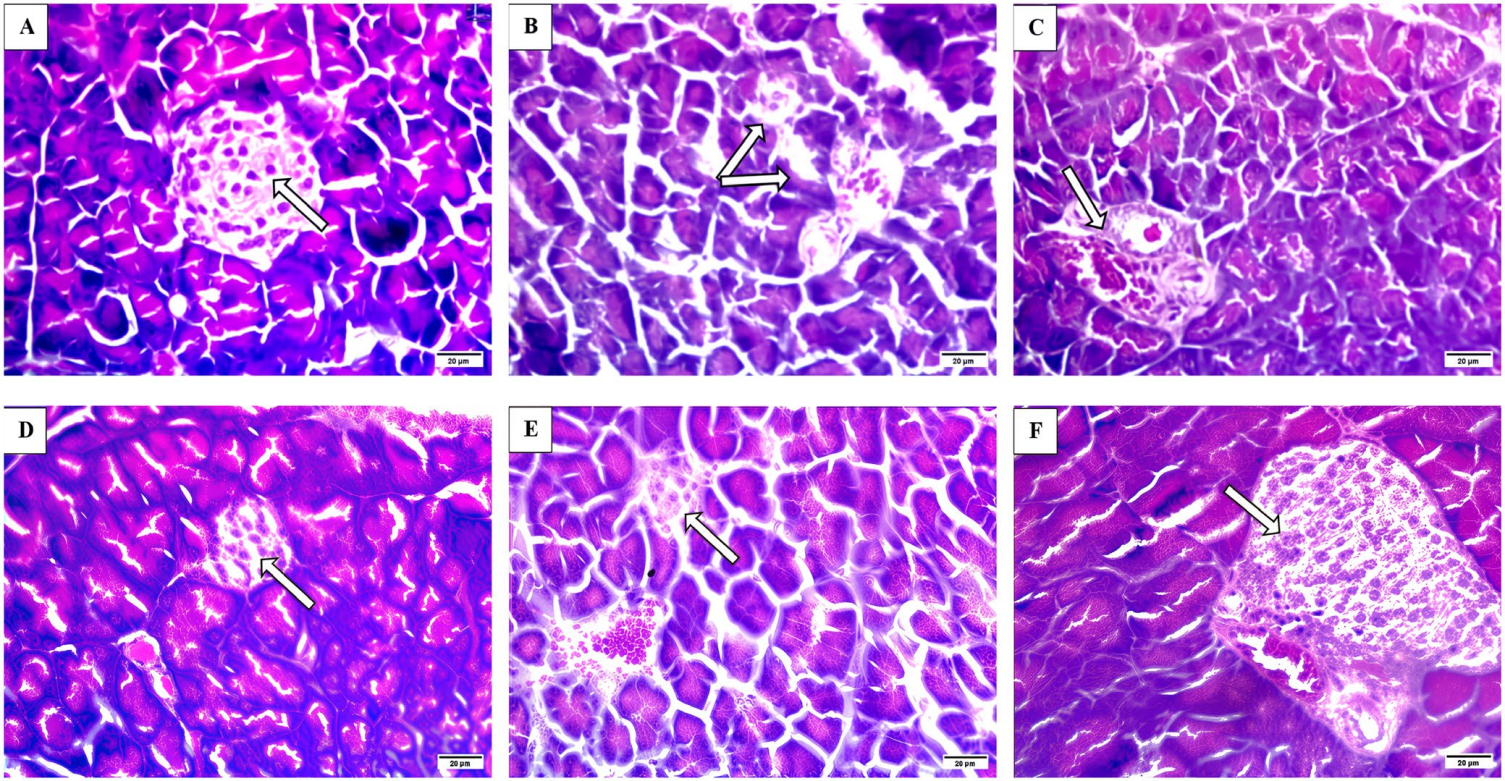
Histology of pancreatic tissue samples of alloxan‐induced diabetic mice. (A) Normal control group—the arrow represents relatively preserved islets of the pancreas; (B) diabetic control group—the arrow points to the atrophied, reduced clusters of pancreatic islet cells; (C) positive control group—the arrow marks the improved pancreatic islet morphology that was previously damaged by disease progression; (D) low dose group—the arrow highlights pancreatic islets that have partially improved; (E) medium dose group—the arrow highlights pancreatic islets that have undergone partial improvement; (F) high dose group—the arrow marks pancreatic islet cells that have been relatively preserved after being damaged by disease progression.

The histology of the kidney (Figure 6) revealed that the normal control group exhibited relatively preserved renal architecture with identifiable glomerular and tubular structures. In diabetic kidneys, the renal architecture appeared altered, with prominent cytoplasmic vacuolation—a feature less prominent in the normal control group. The glibenclamide treated group showed improved renal architecture with identifiable glomerular structures, whereas the polyherbal formulation‐treated groups exhibited partial to relatively preserved renal architecture and a reduction in cytoplasmic vacuolation compared with the diabetic control group.

**FIGURE 6:**
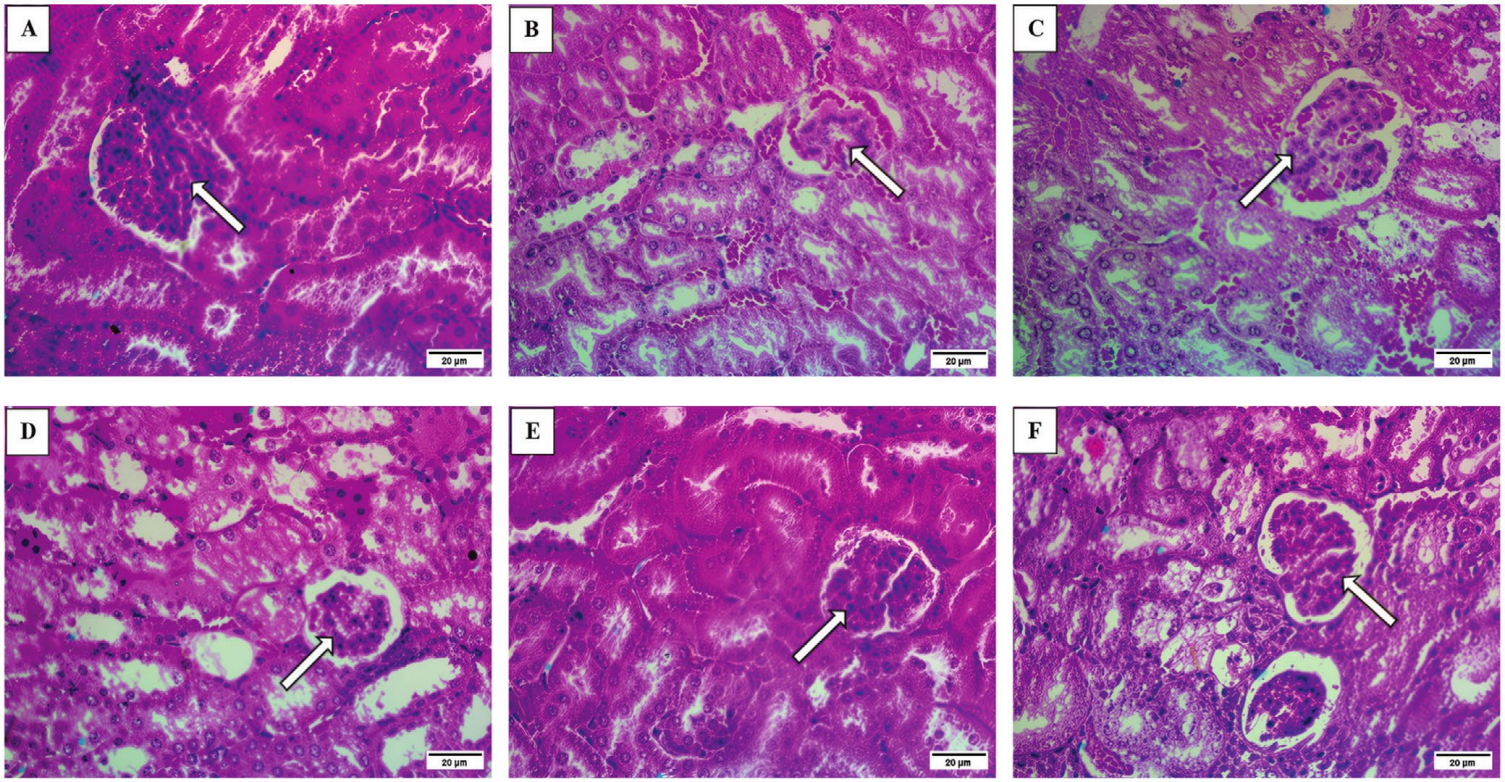
Histopathological analysis of kidney tissue. (A) Normal control group—the arrow highlights relatively preserved renal architecture with identifiable glomerular and tubular structures; (B) diabetic control group—the arrow shows an altered renal architecture with prominent cytoplasmic vacuolation; (C) positive control group—the arrow points to an improved renal architecture with identifiable glomerular structures and less prominent cytoplasmic vacuolation; (D) low dose group—the arrow indicates a partial recovery in renal architecture; (E) medium dose group—the arrow similarly marks a partial improvement in renal cell organization; (F) high dose group—the arrow denotes a relatively preserved renal architecture along with a reduction in cytoplasmic vacuolation.

In the diabetic liver tissue, hepatocytes were swollen with scattered areas of focal necrosis. In contrast, the normal control group exhibited a relatively preserved arrangement of cells, free from disease‐related changes. The glibenclamide‐treated group showed restoration of the improved liver architecture. In the polyherbal formulation‐treated groups, histopathological examination revealed partial improvement in the hepatic architecture at the low dose and partial recovery of liver cell organization at the medium and high doses compared with the diabetic control group (see Figure 7).

**FIGURE 7:**
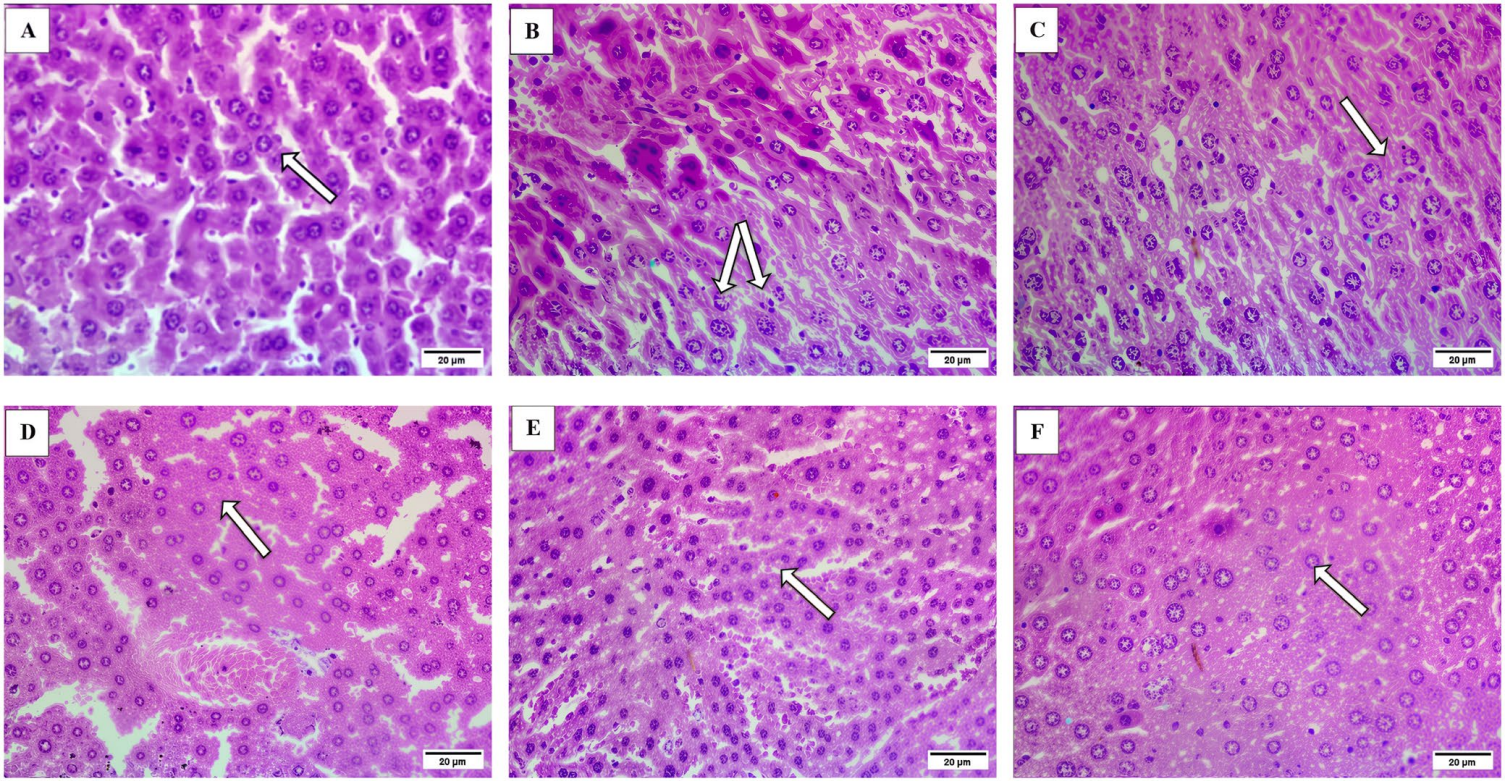
Histopathological analysis of liver tissue. (A) Normal control group—the arrow highlights the orderly, well‐organized arrangement of the liver cells; (B) diabetic control group—the arrow points to swollen liver cells exhibiting focal necrosis; (C) positive control group—the arrow reveals an improved, organized structure; (D) low dose group—the arrow indicates a partial improvement of the liver cell architecture; (E) medium dose group—the arrow shows a similar partial recovery in the arrangement of the liver cells; (F) high dose group—the arrow demonstrates that the liver cells have undergone partial structural recovery.

## 4 Discussion

Diabetes mellitus is a multifactorial metabolic condition in which persistent hyperglycemia, oxidative stress, inflammation, pancreatic β‐cell dysfunction, and dysregulated lipid metabolism collectively contribute to disease progression and its associated complications (Ahmed and Manirujjaman 2026). Although currently available antidiabetic therapies are effective in improving glycemic control, their prolonged use may be limited by adverse outcomes, incomplete protection against diabetes‐associated complications, and accessibility challenges in many resource‐limited settings (Ansari et al. 2024; Ahmed and Manirujjaman 2026). Therefore, there is growing interest in developing plant‐based therapeutic strategies able to simultaneously target multiple pathological mechanisms involved in diabetes.

Building upon our previous comparative in vitro evaluation of five medicinal plants (Ahmed, Hasan, Ahmmed, et al. 2025), the present study developed a rationally designed polyherbal formulation [(AP)_2_M] and investigated its biological potential through comprehensive in vitro and in vivo assessments. The formulation demonstrated appreciable polyphenolic content together with antioxidant and carbohydrate‐digesting enzyme inhibitory activities, exhibited a preliminary toxicity profile, and produced marked antihyperglycemic and antihyperlipidemic effects in type 1 diabetic mice. Improvements in biochemical parameters were further supported by histopathological observations in the pancreas, liver, and kidneys. Collectively, these findings suggest that the formulation successfully translated the complementary biological properties identified during the preliminary comparative screening into measurable therapeutic effects in a diabetic mouse model.

Methanol was chosen as the solvent for extracting the polyherbal mixture due to its efficiency in isolating bioactive compounds, while maceration was employed to optimize yield (Lee et al. 2024). Phytochemicals identified in plants have been linked to their antioxidant, antidiabetic, and antihyperlipidemic properties. It has been demonstrated that they effectively scavenge free radicals and contribute to their potential therapeutic effects (Akpoveso et al. 2023). By neutralizing oxidative stress, these bioactive compounds help prevent lipid peroxidation, which is a key factor in diabetes‐related complications, including hyperlipidemia and cardiovascular diseases (Valgimigli 2023). In recent years, extensive research has highlighted the medicinal properties of plant‐derived antioxidants in managing metabolic conditions, reinforcing the relevance of polyherbal formulations in diabetes treatment (Akpoveso et al. 2023).

Phytochemical results demonstrated that the polyherbal formulation possessed a substantial level of phenolics and flavonoids (111.52 ± 0.444 mg of GAE/g and 211.30 ± 5.129 mg of CAE/g, respectively) (Table 1). These findings are comparable to those reported for other medicinal plants recognized for their potent antioxidant properties, such as *Rosmarinus officinalis* and *Quercus faginea* (Bejenaru et al. 2024; Ferreira et al. 2018). Phenolics and flavonoids are well recognized for their free radical scavenging activity and are considered major contributors to the antioxidant potential of medicinal plants (Pannucci et al. 2023). The antioxidant efficacy of the formulation was further validated through DPPH and metal chelating assays. These assays are widely employed in scientific studies to assess antioxidant potential (Bibi Sadeer et al. 2020).

The polyherbal formulation exhibited 71.18% ± 0.521% inhibition at 200 μg/mL, which, although slightly lower than AA (84.90% ± 0.408%), indicates a concentration‐dependent strong free radical scavenging activity. Similar findings were reported for polyphenol‐rich extracts from eight medicinal plants (Checkouri et al. 2020), which exhibited a comparable concentration‐dependent DPPH radical scavenging effect. Additionally, the MCA assays of the polyherbal formulation (74.99% ± 0.751%) suggest its potential to counteract metal‐induced oxidative stress, further supporting its antioxidant potential. This is consistent with previous studies by Shanmugasundaram et al. (2016).

Several studies highlight the impact of α‐amylase and α‐glucosidase inhibitors in managing hyperglycemia (Nisar et al. 2022; Poovitha and Parani 2016; Zhang et al. 2022). α‐Amylase, present in saliva, pancreatic secretions, and several other tissues, catalyzes the breakdown of complex carbohydrates into dextrin and maltose, contributing to postprandial hyperglycemia (Kaur et al. 2021). In contrast, α‐glucosidase, located in the small intestine, also hydrolyzes carbohydrates to glucose (Dirir et al. 2022). Inhibition of both enzymes slows carbohydrate digestion, reducing postprandial blood glucose concentrations (Gong et al. 2020). Given their significance in diabetes treatment, researchers are exploring natural plant‐based products as potential inhibitors of these enzymes (Bashkin et al. 2021).

The polyherbal formulation significantly inhibited these key carbohydrate‐hydrolyzing enzymes, with α‐amylase inhibition at 58.28% ± 0.511% and α‐glucosidase inhibition at 82.53% ± 0.470% at 250 μg/mL. These results align with Picot et al.'s (2014) findings on traditional antidiabetic plants, which exhibited enzyme‐inhibitory activities. Importantly, the moderate inhibition of α‐amylase compared to α‐glucosidase is advantageous, as excessive α‐amylase inhibition is often linked to gastrointestinal discomfort (Tundis et al. 2010).

The oral acute toxicity assessment in mice is commonly employed in pharmacological and toxicological studies to evaluate the safety and potential risks of compounds—including plant extracts, synthetic drugs, and natural products—prior to proceeding to more complex in vivo investigations (OECD 2002). It provides a straightforward and rapid means of evaluating the harmful effects of substances following a single oral dose. This test helps determine the toxicity levels, behavioral changes, and possible lethal dose (LD_50_) of the compound in mice, providing valuable insights into its safety profile (OECD 2002; Chinedu et al. 2013).

The acute toxicity assessment of the formulation revealed an LD_50_ of 900 mg/kg body weight, indicating a moderate safety profile. This LD_50_ value falls within GHS Category 4 (300–2000 mg/kg) according to the OECD 423 acute oral toxicity classification criteria, suggesting moderate acute oral toxicity (OECD 2002). During the experiment, mild clinical signs—including reduced food intake, behavioral changes, and diarrhea—were observed at higher doses with dose‐dependent mortality. In contrast, no signs of acute toxicity, mortality, or severe behavioral abnormalities were observed among the tested doses for the in vivo antidiabetic study. These findings are consistent with previous studies on polyherbal formulations used in ethnomedicine (Sholikhah et al. 2020; Gopalakrishna Pillai et al. 2017).

Based on the acute toxicity assay, three doses (30, 60, and 120 mg/kg body weight) were selected for further evaluation in alloxan‐induced diabetic mice. The alloxan‐induced diabetic model is widely used for the preliminary evaluation of antidiabetic agents because it enables assessment of their ability to attenuate hyperglycemia and associated metabolic disturbances under conditions of oxidative stress and pancreatic β‐cell injury (Lenzen 2008). Alloxan triggers diabetes by generating ROS that swiftly damage pancreatic β‐cells, resulting in insulin deficiency and hyperglycemia. In this study, diabetic mice receiving the polyherbal extract demonstrated a marked dose‐dependent reduction (*p* < 0.001) in fasting‐state blood glucose. The effective doses observed are comparable to those demonstrated in prior studies (Arabnozari et al. 2024; Amare et al. 2022; Madić et al. 2021), in which similar doses have been widely studied for their antihyperglycemic properties.

Diabetes is often associated with a significant loss of body weight resulting from excessive muscle breakdown and fat depletion (Hebert and Nair 2010). Oral administration of the polyherbal formulation led to a notable increase in body weight in comparison with diabetic control mice, indicating an improvement in the diabetes‐associated loss of body weight. A similar weight‐restoring effect of a polyherbal formulation was observed in the study conducted by Alhamhoom et al. (2023).

Hyperglycemia is also linked to elevated lipid biomarkers such as TG, TC, LDL, and VLDL, accompanied by decreased HDL levels (Dagnew et al. 2021). In this experiment, treatment with the polyherbal formulation and glibenclamide led to a marked reduction in lipid biomarkers while increasing HDL levels, consistent with the findings reported by Rahman et al. (2022) and Amare (2021). According to phytochemical data, the polyherbal formulation contains a significant level of polyphenol contents (phenolic and flavonoid). These compounds are widely known for their antioxidant and lipid‐modulating properties (Wei et al. 2025). Previous studies suggest that plant polyphenols may influence lipid homeostasis through several mechanisms, including direct modulation of lipid metabolism, attenuation of oxidative stress, regulation of mitochondrial function, and alleviation of inflammation (Wei et al. 2025). These activities may contribute to improved lipid metabolism and could partly explain the reductions in TG, TC, LDL, and VLDL observed following treatment with the polyherbal formulation. Although the precise mechanisms underlying the observed hypolipidemic effects were not assessed in the present study, these findings may reflect the combined biological activities of these phytochemicals.

Liver function biomarkers, such as SGOT and SGPT, are often elevated in diabetes due to hepatic damage (Haffar et al. 2021). The polyherbal extract effectively reduced SGPT and SGOT levels, suggesting hepatoprotective effects. These findings are consistent with those presented by Alhamhoom et al. (2023) and Aljazzaf et al. (2023), which indicated similar protective actions on liver health in diabetic models.

Histopathological analysis further supports the therapeutic potential of the polyherbal formulation. Pancreatic tissue examination revealed improved preservation of the islets of Langerhans, suggesting protection against alloxan‐induced oxidative damage. Additionally, the liver cells exhibited partial recovery of their cellular architecture, suggesting hepatoprotective effects. The kidney tissue also showed signs of recovery, as evidenced by the improved renal architecture, along with a reduction in cytoplasmic vacuolation. This is consistent with studies by Rahman et al. (2022), which have indicated β‐cell protective effects due to their polyphenol‐rich compositions. The histological findings corroborate the biochemical data, suggesting that the observed antidiabetic effects may involve multiple biological processes, including antioxidant activity, inhibition of carbohydrate‐hydrolyzing enzymes, and protection of pancreatic, renal, and hepatic tissues (Madić et al. 2021).

Overall, the polyherbal formulation demonstrated significant antidiabetic effects at tested doses (30, 60, and 120 mg/kg), with strong statistical support (*p* < 0.001). Compared with previous reports in which individual extracts of *P. emblica* , *P. nigrum* , *A. marmelos* , *M. oleifera* , and *A. indica* generally required higher doses (> 200 mg/kg) to achieve comparable antihyperglycemic effects (Lin et al. 2024; Olayaki et al. 2015; Satyanarayana et al. 2015; Upadhya et al. 2004), the present formulation showed antidiabetic effects at lower tested doses. While differences in experimental conditions between studies may limit direct comparisons, this finding may reflect the complementary actions of the constituent plant extracts.

### 4.1 Limitations and Future Directions

Although the current study offers initial evidence supporting the antidiabetic and antihyperlipidemic potential of the novel polyherbal formulation [(AP)_2_M], some limitations should be acknowledged. The phytochemical characterization was limited to total phenolic and flavonoid contents without compound‐level profiling (e.g., HPLC, LC–MS, or GC–MS), and the molecular mechanisms underlying the observed biological effects were not directly investigated. In addition, although the formulation demonstrated efficacy at relatively low doses, the potential synergistic interactions among its constituent plant extracts were not specifically evaluated. Future studies should therefore focus on comprehensive phytochemical characterization, mechanistic investigations, synergy assessments, and long‐term safety evaluations to further validate these preliminary findings and support the development of this formulation as a potential therapeutic candidate for diabetes and associated dyslipidemia.

## 5 Conclusion

The present study provides preliminary evidence supporting the antioxidant, antihyperglycemic, and antihyperlipidemic potential of the novel polyherbal formulation [(AP)_2_M]. The formulation demonstrated significant dose‐dependent improvements in blood glucose levels, lipid profile, liver function biomarkers, and histopathological alterations in alloxan‐induced diabetic mice. At the highest tested dose, the formulation exhibited antihyperglycemic effects comparable to those of glibenclamide. Collectively, these findings suggest that the complementary actions of the constituent medicinal plants may contribute to the observed therapeutic effects of [(AP)_2_M], highlighting its potential as a promising polyherbal formulation for managing diabetes and its associated metabolic complications. Nevertheless, further studies involving comprehensive phytochemical characterization, mechanistic investigations, evaluation of the complementary interactions among the constituent plant extracts, and long‐term safety and efficacy assessments are warranted to validate these preliminary findings and support its future therapeutic development.

## Author Contributions

**Md. Shakil Ahmed:** conceptualization, methodology, software, investigation, data curation, formal analysis, visualization, writing – original draft. **Md. Taohid Hasan:** investigation, writing – review and editing. **Md. Bismillah:** investigation, formal analysis, writing – review and editing. **M. Matiar Rahman:** conceptualization, supervision, validation, project administration, resources, writing – review and editing. **Mohammad Ismail Hossain:** conceptualization, methodology, resources, data curation, validation, writing – review and editing. **Masudul Hasan Khan:** conceptualization, data curation, validation, writing – review and editing. **Mohammad Amirul Islam:** conceptualization, methodology, resources, writing – review and editing.

## Funding

The authors have nothing to report.

## Ethics Statement

This study was approved by the Institutional Animal, Medical Ethics, Bio‐Safety, and Bio‐Security Committee (IAMEBBC) for experiments involving animals, humans, microbes, and living natural sources, under the approval memo no: 38/320/(47)IAMEBBC/IBSc, Institute of Biological Sciences, University of Rajshahi, Bangladesh.

## Conflicts of Interest

The authors declare no conflicts of interest.

## Supporting information


**Appendix S1:** fsn372520‐sup‐0001‐AppendixS1.docx.

## Data Availability

The data that support the findings of this study are available from the corresponding author upon reasonable request.
