# Supplementary material for: Therapeutic Potential of [(AP) 2M]: A Rationally Designed Polyherbal Formulation for Antidiabetic and Antihyperlipidemic Effects in Alloxan‐Induced Diabetic Mice
Source: Food Sci Nutr. 2026 Oct 26;14(11):e72520. doi: 10.1002/fsn3.72520 (PMC13710797; doi:10.1002/fsn3.72520)
Supplement: Supplementary file 1 [file FSN3-14-e72520-s001.docx]

**Supplementary Materials**

1. **Polyherbal Formulation Proportion Calculation:**

**Inverse-weighting formula**

Because plant materials with lower total polyphenolic content (TPC) were assigned higher proportions in the formulation, an inverse-weighting approach was used:

*w_i_ =* $\frac{1}{{\sqrt[4]{S}}_{TPC, i}}$

$$P_{i}(\%)=\frac{w_{i}}{{\sum w}_{j}}\times100$$

Here,

*i =* Sample index

*j =* Summation index

*w_i =_* inverse weight for sample *i*

S_TPC,_ *_i_* = TPC score for sample *i*

*P_i_ =* normalized mass proportion of sample *i*

| **Sample** | ***^a^*TPC (mg GA/g)** | ${\sqrt[\boldsymbol{4}]{\boldsymbol{S}}}_{\boldsymbol{TPC}}$ | ***w_i_*** | $\boldsymbol{\sum w}_{\boldsymbol{j}}$ | $\boldsymbol{P}_{\boldsymbol{i}}\boldsymbol{(\%)}$ | ***^b^*Final proportion (%)** |
| --- | --- | --- | --- | --- | --- | --- |
| *P. emblica* | 196.09 | 3.742 | 0.267 | 1.702 | 15.70 | 17.70 |
| *A. marmelos* | 69.14 | 2.883 | 0.347 |  | 20.38 | 20.38 |
| *P. nigrum* | 26.42 | 2.267 | 0.441 |  | 25.92 | 23.92 |
| *M. oleifera* | 93.48 | 3.109 | 0.322 |  | 18.90 | 18.90 |
| *A. indica* | 89.49 | 3.076 | 0.325 |  | 19.10 | 19.10 |
| TPC: Total phenolic content. | | | | | | |

**Note that:**

a. The TPC values used for the formulation calculation were obtained from an independent experimental determination.
b. Based on the normalized inverse fourth-root weighting calculation, the initial proportion of *P. nigrum* was 25.92%, while that of *P. emblica* was 15.70%. Because *P. nigrum* showed comparatively higher toxicity in the brine shrimp lethality assay, its proportion was reduced by 2.00 percentage points, from 25.92% to 23.92%. The corresponding 2.00 percentage points were transferred to *P. emblica*, which showed the comparatively lowest toxicity among the five plant materials, increasing its proportion from 15.70% to 17.70%. This limited adjustment was made as a safety-oriented modification while retaining the TPC-derived proportions of the other three plant materials. Overall, the purpose of this approach was to achieve a balanced contribution of all five plant materials while preventing a plant with relatively high polyphenolic content from disproportionately dominating the formulation.
2. **LD₅₀ Calculation for Acute Oral Toxicity Assessment**

| Dose (mg/kg) | Total mice per group | Mortality |
| --- | --- | --- |
| 100 | 5 | 0 |
| 200 |  | 0 |
| 400 |  | 1 |
| 800 |  | 2 |
| 1600 |  | 5 |

The arithmetic equation of Karber for LD₅₀:

LD₅₀ = LD_100_ − $\frac{\sum(a\times b)}{n}$

Here,

LD_100_ =lowest dose producing 100% mortality = 1600

a = dose interval = (100, 200, 400, and 800)

b = average mortality between adjacent doses = (0, 0.5, 1.5, 3.5)

*n* = number of animals in each group = 5

LD₅₀ = 1600 − $\frac{\{(100 x 0) + (200 x 0.5) + (400 x 1.5) + (800 x 3.5\}}{5}$ = 900 mg/kg

The LD_50_ is approximately **900 mg/kg**.

3. **Tables for the In Vivo Antidiabetic Experiment**

| **Table S1:** The effect of herbal formulation on the blood glucose levels of alloxan-induced diabetic mice. | | | | | |
| --- | --- | --- | --- | --- | --- |
| Groups | Blood Glucose Levels (mg/dL) | | | | |
|  | Before Treatments Start | Day-1 | Day-7 | Day-14 | Day-21 |
| Normal | 99.3 ± 4.47 | 99.9 ± 3.37 | 101.7 ± 2.96 | 100.5 ± 3.10 | 100.2 ± 2.18 |
| Diabetes | 102.3±1.35 | 241.8 ± 4.65 | 279.3± 6.96 | 306.3± 5.51 | 342.6 ± 9.23 |
| Glibenclamide | 100.2 ± 3.89 | 240.0 ± 11.25 | 208.2± 4.36 | 167.7 ± 3.49 | 147.6 ± 4.26 |
| Low dose | 96.6 ± 1.86 | 246.0 ± 3.15 | 207.6 ± 5.05 | 186.3 ± 1.89 | 171.3 ± 6.48 |
| Medium dose | 102.3 ± 4.89 | 243.9 ± 3.73 | 202.2 ± 6.10 | 175.5 ± 6.00 | 160.2± 7.02 |
| High dose | 103.8 ± 6.20 | 241.5 ± 10.33 | 202.2 ± 3.89 | 165.9 ± 3.67 | 147.3± 5.96 |
| Values are reported as mean ± SD, with *n* = 6 mice per group. | | | | | |

| **Table S2:** Impact of the polyherbal formulation on bodyweight changes in alloxan-induced diabetic mice. | | | | | |
| --- | --- | --- | --- | --- | --- |
| groups | Body weight (g) | | | | |
|  | Before Treatment Start | Day-1 | Day-7 | Day-14 | Day-20 |
| Normal | 26.8 ± 1.17 | 28.2 ± 1.94 | 30.5 ± 2.43 | 31.3 ± 2.88 | 32.7 ± 2.66 |
| Diabetes | 29.0 ± 1.41 | 27.5 ± 0.55 | 26.3 ± 0.82 | 25.0 ± 1.58 | 24.0 ± 0.82 |
| Glibenclamide | 29.2 ± 0.98 | 27.5 ± 1.05 | 29.8 ± 0.98 | 31.3 ± 1.21 | 33.0 ± 1.26 |
| Low dose | 27.3 ± 1.21 | 25.5 ± 1.05 | 26.8 ± 0.75 | 28.3 ± 1.21 | 30.5 ± 1.05 |
| Medium dose | 28.5 ± 1.05 | 26.8 ± 1.17 | 28.2 ± 0.75 | 30.0 ± 0.89 | 31.8 ± 1.17 |
| High dose | 28.7 ± 0.82 | 27.2 ± 1.17 | 28.5 ± 1.05 | 30.2 ± 1.17 | 32.7 ± 1.03 |
| Values are reported as mean ± SD, with *n* = 6 mice per group. | | | | | |
